# *PLOS Medicine* 2017 Reviewer and Editorial Board Thank You

**DOI:** 10.1371/journal.pmed.1002550

**Published:** 2018-03-15

## Abstract

PLOS and the *PLOS Medicine* team would like to express our appreciation to the academic editors, guest editors, and reviewers who contributed to the peer-review process in 2017.

PLOS and the *PLOS Medicine* team would like to express our appreciation to the academic editors, guest editors, and reviewers who contributed to the peer-review process this past year. We are indebted to our volunteers who generously give their time and expertise to thoroughly review research and advance Open Access. In 2017, *PLOS Medicine* received the help of 119 Editorial Board members and 48 guest editors to evaluate 1,865 submissions. Combined with the participation of 861 reviewers, these efforts enabled us to publish 166 research articles at a high standard of novelty, methodological validity, and transparency in reporting.

The names of our Editorial Board members and guest editors who handled submitted manuscripts in 2017 appear in the Supporting Information as S1 Editor List and as S1 Guest Editor List. Our reviewers appear in the Supporting Information as S1 Reviewer List. Please accept our gratitude for your dedicated support of *PLOS Medicine* and our efforts to promote Open Science, making research on human health available to all without restriction. We thank you all.

## Supporting information

S1 Editor List Alphabetical list of 2017 *PLOS Medicine* Editorial Board Members. (PDF)

S1 Guest Editor List Alphabetical list of 2017 *PLOS Medicine* Guest Editors. (PDF)

S1 Reviewer List Alphabetical list of 2017 *PLOS Medicine* peer reviewers. (PDF)
