# Supplementary material for: PLOS Medicine 2017 Reviewer and Editorial Board Thank You
Source: PLoS Med. 2018 Mar 15;15(3):e1002550. doi: 10.1371/journal.pmed.1002550 (PMC5854231; doi:10.1371/journal.pmed.1002550)
Supplement: S1 Editor List — (PDF) [file pmed.1002550.s001.pdf]

PLOS | MEDICINE

PLOS Medicine would like to thank all those who served on the Editorial Board in 2017:

Marcus Altfeld
Elizabeth A. Ashley
Sanjay Basu
James G. Beeson
Chris Beyrer
Zulfiqar A. Bhutta
Carol Brayne
James Derek Brenton
Karim Brohi
Peter Byass
Suzanne C. Cannegieter
Lucy C. Chappell
George Davey Smith
Louisa Degenhardt
Matthias Egger
Carlton Evans
Paul Garner
Elvin Hsing Geng
Davina Ghersi
Jeremy D. Goldhaber-Fiebert
Manuel B. Graeber
Rebecca Freeman Grais
Ed Gregg
Simon Hales
Timothy Hallett
Charlotte Hanlon
Stephan Harbarth
David Harlan
Phillipa J. Hay
Suzanne Hill
Tom Huizinga
Lars Hviid
John P. A. Ioannidis
Rachel Jewkes
Ricky W. Johnstone
Aaron S. Kesselheim
Noah Kiwanuka
Keith P. Klugman
Mirjam E. E. Kretzschmar
Sanjeev Krishna
Margaret E. Kruk
Emmanuel Lagarde
Claudia Langenberg
Tze Kin Lau
Kelley Lee
Susan Lewallen
Cathryn Lewis
Marc Lipsitch
Nicola Low
David S. Ludwig
Ronald C. W. Ma
Malcolm R. Macleod
Elaine Mardis
Mark I. McCarthy
Clara Menendez
David Menon
Anne Merriman
John Metcalfe
Philippa Middleton
Bruce Miller
Cosetta Minelli
Lynne Meryl Mofenson
David Moher
Suerie Moon
Megan Murray
R. Srinivasa Murthy
Jenny E. Myers
Marie-Louise Newell
Olivier Neyrolles
Christelle Nguyen
Abdisalan Mohamed Noor
David Osrin
Matthew J. Page
Madhu Pai
Anushka Patel
Vikram Patel
David Peiris
Malik Peiris

Lars Ake Persson
Barry M. Popkin
Andrew Prentice
Kazem Rahimi
Don A. Redelmeier
Andrew Rice
Stephen John Rogerson
Sydney Rosen
Joshua A. Salomon
Peter Sandercock
Stephanie L. Sansom
Mathuram Santosham
Martin Schreiber
Stefan Schreiber
Jaime Sepulveda-Amor
Barbara Shacklett
Steve D. Shapiro
Aziz Sheikh
Nandi Siegfried
Amit Singal
Mervyn Singer
Peter Singer
Gordon C. Smith
Thomas A. Smith
Ebgert Sondorp
Zirui Song
David Stuckler
Amitabh Bipin Suthar
Maarten W. Taal
Mark Tomlinson
Alexander C. Tsai
James K. Tumwine
Patrick Vallance
Jean-Louis Vincent
Lorenz von Seidlein
Theo Vos
Sheri D. Weiser
Nicholas J. White
Joshua Z. Willey
Linda Wright
Wei Zheng
