# Supplementary material for: PLOS Medicine 2017 Reviewer and Editorial Board Thank You
Source: PLoS Med. 2018 Mar 15;15(3):e1002550. doi: 10.1371/journal.pmed.1002550 (PMC5854231; doi:10.1371/journal.pmed.1002550)
Supplement: S1 Guest Editor List — (PDF) [file pmed.1002550.s002.pdf]

PLOS Medicine would like to thank all those who served as Guest Academic Editors in 2017:

Garnet Anderson
Linda-Gail Bekker
Tom Boyles
Lutz P. Breitling
Nathalie Broutet
Simon Capewell
Mike Clarke
Silvia De Sanjosé
Steven G. Deeks
Luis A. Diaz
Nicholas M. Fisk
Adriane Fugh-Berman
Raquel C. Gardner
Herman Goossens
Wayne D. Hall
Alastair D. Hay
Jacob Hughey
Marc Ladanyi
Carolyn S. P. Lam
Bruce P. Lanphear
Debbie A. Lawlor
Michael Lean
William Ledger
Justin Lessler
Sharon R. Lewin
Sherene Loi
Michelle M. Mielke
John D. Minna
Lynne Meryl Mofenson
Ivo Mueller
Bruce Neal
David Ogilvie
Holly Gwen Prigerson
Martin J. Prince
David Rees
Jurgen Rehm
Giuseppe Remuzzi
Andrew S. C. Rice
Steven Riley
Naveed Sattar
Steven R. Steinhubl
O. Colin Stine
S. V. Subramanian
Cecile Viboud
Nicholas J. Wareham
Rudi G. J. Westendorp
Sarah H. Wild
Clifford J. Woolf
