# Supplementary material for: PLOS Medicine 2017 Reviewer and Editorial Board Thank You
Source: PLoS Med. 2018 Mar 15;15(3):e1002550. doi: 10.1371/journal.pmed.1002550 (PMC5854231; doi:10.1371/journal.pmed.1002550)
Supplement: S1 Reviewer List — (PDF) [file pmed.1002550.s003.pdf]

PLOS | MEDICINE

PLOS Medicine would like to thank all those who reviewed on behalf of the journal in 2017:

Salim Abdool Karim
Frances Aboud
Shafika Abrahams-Gessel
Laith Abu-Raddad
Hans Ackerman
Kristina Adachi
Jean Adams
Peter Adamson
Xabier Agirre
Kawango Agot
Aminul Ahmed
Marcella Alsan
Christian Althaus
Benjamin Althouse
Doug Altman
Mary Amato
Soter Ameh
Craig Anderson
Cheryl Anderson
Peter Andrews
Gavin Andrews
Jeannie Annan
Kaarin Anstey
Tomas Aragon
Shams Arifeen
Kenneth Ataga
Jared Baeten
David R. Baldwin
Piotr Bandosz
David Bangsberg
Kathleen Banks
Stephen Barclay
Ruanne Barnabas
Michael Barnes
Deborah Barnes
Scott Barnhart
Alison Bateman-House
Shannon Bates
David Bates
Rachel Batterham
Jasper Been
Katy Bell
Eran Bendavid
Phillip Bennett
Rhonda Bentley-Lewis
Howard Bergman
Benjamin Berman
Rameen Beroukhim
David Berrigad
Ana Pilar Betrán
Kavi Bhalla
Nita Bhandari
Achuyt Bhattarai
Raj Bhopal
Peter Biberthaler
Barbara Bierer
Timothy Billiar
Charles Billington
Laurent Billot
Petter Bjornstad
Mairead Black
Deborah Blacker
Tony Blakely
Joel Blankson
Hannah Blencowe
David Blumenthal
Isaac Bogoch
Michael Boivin
Maureen Bolon
Nigel Bolster
Peter Boone
Andrew Booth
Jacob Bor
Erika Borella
Cesar Borlongan
Annick Borquez
Jaime Bosch
Nabila Bouatia-Naji

Barbara Boucher
Mark Boyd
Tom Boyles
Fiona Bragg
Vlasios Brakoulias
George Bray
Simon Brooker
Allen Brown
Colin Brown
Jens Ivar Brox
Jessie Brunner
Richard Bryant
Ashley Bryce
Yvonne Bryson
Radek Bukowski
Frank Buntinx
Stephen Burgess
Helen Bygrave
Felipe Cabello
Caroline Cameron
Francisco Campos
Simon Capewell
Marly Cardoso
Michael Carey
Adam Carrico
Jennifer Carter
Simon Cauchemez
Jonathan Caulkins
Connie Celum
Amitabh Chak
Larry Chang
Jorge Chavarro
Hannah Cheeseman
Ken Chen
Alice Chen-Plotkin
Jin-Ho Choi
Lumbwe Chola
Mickey Chopra
Joan Chow
Ingrid Elisabeth Christophersen
Kathryn Chu
Andrea Ciaranello
Andrea Cipriani
David Clarke
Robert Clarke
Chuck Cleland
Kate Clouse
Sean Coffey
Patricia Coffey
Joyce Coker
Tim Colbourn
Matthew Coldiron
Stephen Cole
Yvonne Commodore-Mensah
Cris Constantinescu
Matthew Cooperberg
Christine Cooper-Vince
Carol Coupland
Martin Cowie
N. Crepaz
Colin Crooks
Casey Crump
Liwang Cui
Pim Cuijpers
William Culpepper
Coleen Cunningham
Judith Currier
Adnan Custovic
Francois Dabis
Goodarz Danaei
Karen Daniels
Dawood Darbar
Nicole Darmon
Sumona Datta
Mike Daube
Ross Davenport
Mary-Ann Davies
Daniel Davis
Elizabeth Dawson-Hahn
Felix Day
Richard Daynard
Silvia de Sanjosé
Dennis Deapen
Michele Decker
Joost Dekker
Cinzia Del Giovane
Stefano Del Prato
Pierre Delanaye

Paolo Deluca
Nikos Demiris
Martin Denvir
Rahul Deo
Michael Dewey
Kathryn Dewey
Mario Di Napoli
Ramon Diaz-Arrastia
Karla Diaz-Ordaz
Xavier Ding
Josie Dixon
Roger Dixon
Bruce Dobkin
Daniela Dobre
Raphael Dolin
Sidsel Domazet
Esteban Domingo
Deborah Donnell
Christl Donnelly
Gail D'Onofrio
Jenny Donovan
Abdel Douiri
David Dowdy
Charles Drake
Brian Draper
Shufa Du
Stephen Duffy
Patrick Duffy
Carole Dufouil
Radboud Duintjer Tebbens
Shanta Dutta
Terrence Dwyer
Mark Dybul
Tobias Eckle
Andrew Edmonds
Donald Edmondson
Jennifer Edwards
Philip Efron
Michael Eikmans
Kristina Elfving
Adam Elshaug
Holger Eltzschig
Jonathan Emberson
Jonathan Emens
Brinda Emu
Ingunn Engebretsen
Maijaliisa Erkkola
Katharine Esselen
Anne Fanning
Bart Fauser
Giovanni Andrea Fava
Maurizio Fava
Seena Fazel
Neelam Feachem
Matthew Feinstein
David Felson
Bart Ferket
Rashida Ferrand
Sarah Fidler
Sarah Finer
Lawrence Fleckenstein
Paul Fleming
Simon Fleminger
Corinne Fligner
Peter Flom
Richard Fluck
Nathan Ford
Nita Forouhi
Russell Foster
Edward Fottrell
Matthew Fox
Betsy Foxman
Emma France
Timothy Frayling
Rachel Freathy
David Fredricks
Sten Fredrikson
Jingyuan Fu
Jennifer Furin
Shyam Gajavelli
Seana Gall
Martin Gallagher
Alison Galvani
Rajesh Gandhi
Giuseppe Gargiulo
Geoff Garnett
Margaret Gatz
Dionne Gesink

Davina Ghersi
Robert Gibbons
Gretchen Gierach
Tom Gift
Anna Gilmore
Malin Gingnell
Richard Glassock
Samuel Goldman
Raúl González-Domínguez
Guy Goodwin
Roland Gosling
Mary Grabowski
Casey Greene
Trisha Greenhalgh
Nicola Greenlaw
Brian Greenwood
Karen Grepin
Marie Griffin
Jamie Griffin
Leif Groop
Sandeep Grover
Yian Gu
Richard Guerrant
Keila Guimaraes
Nicole Guiso
Sumit Gupta
Somya Gupta
Brian Gushulak
Marya Gwadz
Marion Haas
Judith Hahn
Nancy Haigwood
James Hakim
Hakon Hakonarson
Wayne Hall
Margaret Hammerschlag
Johanna Hanefeld
Colleen Hanrahan
Claudia Hanson
Andrea Haqq
Thomas Harrer
Kelly Harvard
Nick Harvey
Zaki Hassan-Smith
Ian Hastings
Jan Hattendorf
Elliott Haut
Diane Havlir
Carol Hawley
Christopher Hawthorne
Alison Hayes
Stanley Hazen
Claire Healy
Renee Heffron
Michal Heger
Robert Heimer
Adel Helmy
Janet Hemingway
Karla Hemming
Walid Heneine
Joshua Herbeck
Amanda Herbert
Ronald Hershow
Frederik Boëtius Hertz
Matthew Hickman
Elizabeth Higgs
Andrew Hill
Martin Hirsch
Jane Hocking
Karen Hoffman
Matthew Hogben
Timothy Hohman
Henne Holstege
Tim Holt
Carol Hopkins Sibley
James Hoxie
Marta Hribal
Yu-Hsiang Hsieh
Jian Hu
Guoqing Hu
Gang Hu
Maria Hughes
Raymond Hui
David Hutton
Emily Hyle
Vincent Iacopino
Mohammad Ikram
Nesrina Imami

Kunihiro Inai
Silvio E. Inzucchi
Collins Iwuji
Tsui J.
Debra Jackson
David Jacobs Jr.
Yves Jacquemyn
Tazeen Jafar
Bryan James
Jan Jansen
Jonathan Jarow
Jonathan Jay
Aurelie Jeandron
Tanya Johns
Samuel Johnson
Leigh Johnson
Wayne Johnson
Jon Jureidini
Claus Kadelka
James Kahn
Andre Kalil
Niranjan Kanesa-Thasan
Mingon Kang
Celeste Karch
Neerja Karnani
Robinson Karuga
Rupert Kaul
Judith Kelsen
Stephen Kennedy
Louise Kenny
Erin Kent
Babajide Keshinro
Hind Khalifeh
Arif Khan
Kiran Khush
Jong-Hoon Kim
Deborah King
Ole Kirk
Mika Kivimäki
Terry Klassen
Eili Klein
Michail Klontzas
Leo Koenderman
Frederick Korley
Roger Kouyos
Naoko Kozuki
John Kraemer
Katharina Kranzer
Kurt Kroenke
Inge Kroidl
Mariana Kruger
Lewis Kuller
Daniel Kuritzkes
Shyama Kuruvilla
Ruth Kutalek
Chris Kypridemos
Niklaus Labhardt
Carl Lachat
Louise Lafortune
Alastair Lamb
Claudio Lanata
Claudia Langenberg
David Langlais
Eric Larson
Warren Laskey
Estrella Lasry-Levy
Bryan Lau
James Lavery
Theresa A. Lawrie
Ha Youn Lee
Kelley Lee
Luke Leenen
Uta Lehmann
Mall Leinsalu
Thomas Leitner
Emmanuelle Leray
Jef Leroy
Justin Lessler
Peter Leth
David Levine
Gary Levy
Gerald Levy
Felicia Lewis
David Lewis
Joseph Lewnard
Joel Lexchin
Xia Liang
Richard Lilford

Benjamin Linas
Bernt Lindtjørn
Sarka Lisonkova
Sarah Little
Wanqing Liu
Li Liu
Bian Liu
Gill Livingston
Nicolas Llosa
Rogerio Lobo
Colin Loftin
Guy Loneragan
Ruth Loos
Janet Lord
Attila Lorincz
Nicola Low
Steven Lubitz
Sebastian Lucas
Jens Lundgren
Peter Lurie
Holly Lynch
Andrew Maas
David Mabey
Stuart MacDonald
James Macinko
Raina MacIntyre
Margaret Mackinnon
Emily MacLean
Freya MacMillan
Peter MacPherson
Kristine Madsen
Matthew Maenner
Laura Magee
Jessica Magidson
Lisa Manhart
Ulrich Mansmann
Jonathan Mant
Mark Mapstone
Nicola Marconi
Ulrich Marcus
Riccardo Marioni
Guy Marks
Jeanne Marrazzo
John Marshall
Glen Martin
Linnet Masese
J. Masjuan
Mhairi Maskew
Rohini Mathur
Alberto Matteelli
Fiona Matthews
Anthony Matthews
Niklas Mattsson
Michael Maze
Pamela Mbabazi
James McCaw
Alex McConnachie
James McCord
James McCormack
Patrick McGann
Rose McGready
Sally McGregor
James McMahon
Barbara Mcpake
Ruth McPherson
Fiona McQuaid
Leah Mechanic
Nicholas Medland
Francis Mégraud
Erik Melén
Edward Melhuish
Werner Mendling
Pauline Mendola
Dick Menzies
Stewart Mercer
Mario Merialdi
Jordi Merino
Sonja Merten
Maria Mesana Graffe
Sarah Meyer
Gesine Meyer-Rath
Jesse Mez
Saskia Middeldorp
Michelle Mielke
Cathy Mihalopoulos
Borislava Mihaylova
Franklin Miller
Elizabeth Miller

Jennifer Miller
Christopher Millett
Edward Mills
Kingston Mills
Adrian Mindel
Lynne Mofenson
David Moher
Vincent Mok
Ali Mokdad
Lyle Moldawer
Zoe Moodie
Kevin Moore
Steven Moore
Sophie Moore
Andrew Moran
Lynn Morris
Jonathan Morrison
Saam Morshed
Melissa Mugambi
Kenneth Mugwanya
Katherine Muldoon
Heather Murray
Angela Mushavi
Victor Mwapasa
Landon Myer
Jenny Myers
Oliver Mytton
Kari Nadeau
Nicolas Nagot
Sue Napierala Mavedzenge
Charles Natanson
Francis Ndowa
J. Craig Nelson
Paul Newcombe
Olivier Neyrolles
Ernest Ng
Shu Wen Ng
Lauren Ng
Nhung Nghiem
Peter Nguhiu
Cliona Ni Mhurchu
Karin Nielsen-Saines
Nobuyuki Nishikiori
Emmanuel Njeuhmeli
Dorte Noeroexe
Christopher Nolan
Peter Nordström
Jane Norman
Bohdan Nosyk
Roberto Novoa
Glen Nowak
Yoshitsugu Obi
Katherine O'Brien
Michelle Odden
Audrey Odom
Michael O'Dwyer
Gina Ogilvie
Lucy Okell
Kelli O'Laughlin
Catherine Oldenburg
Ken Ong
Steven Opal
Jenevieve Opoku
Merete Osler
Nancy Padian
Anne-Laure Page
Daniel Palazuelos
Clovis Palmer
Tae Park
Joel Parker
Omrana Pasha
Sant-Rayn Pasricha
Pau Pastor
Raj Patel
Andrew Paterson
Sumeet Patil
Jon Pedersen
Deborah Persaud
Sanne Peters
Maya Petersen
John Petrie
Mark Petticrew
Paul Pharoah
Jean-Yves Pierga
Duarte Pignatelli
Deenan Pillay
Jean-Francois Pittet
Elizabeth Plimack

Nicola Pocock
Andrew Pollard
Marcus Pond
Chad Porter
Huntington Potter
Andrew Prendergast
Malcolm Price
Timothy Pritts
Charlotte Probst
Shamim Qazi
J. G. Quicke
Kyle Quinn
Ana Quinones
Miriam Rabkin
Atif Rahman
Tanvi Rai
Parminder Raina
Anita Raj
Ambady Ramachandran
Michael Ramharter
Tommy Rampling
Molebogeng Rangaka
Didier Raoult
Andrew Rasmussen
Hannah Ratcliffe
Fahad Razak
Oliver Razum
Mark Reed
David Rees
David Regan
Jurgen Rehm
Nicholas Reich
Christiane Reitz
Andrew Renehan
Susan Resnick
Bharat B. Rewari
Steven Reynolds
Charles Reynolds
Edo Richard
Guy Richards
J. Brent Richards
Kees Rietmeijer
Steven Riley
Beate Ritz
Drucilla Roberts
Courtland Robinson
Leanne Robinson
John Robson
Ana Cecilia Rodriguez
Michael Roerecke
Julio Rojas-Martinez
Megan Romano
Staffan Rosenborg
Philip Rosenthal
Joseph Ross
Giulio Rossi
Daphna Rothschild
Jean-Pierre Routy
Christine Rouzioux
Jamie Rylance
Charumathi Sabanayagam
Caroline Sabin
Jonah Sacha
Greg Sachs
Samy Sadek
Marcel Salathé
Joel Salinas
Matti Sällberg
Emilia Salvadori
Luz Maria Sánchez-Romero
Anna Sandström
Marie Sandvei
John-Paul Sanggaran
Lorenzo Savioli
Mark Schiffman
Amand Schmidt
Ben Schöttker
Matthias Schulze
Gary Schwitzer
W. Secor
Kate Seib
Nicholas Selby
Graham Serjeant
Robert Shafer
William Shafer
Sohrab Shah
Anoop Shah
Holly Shakya

Marian Shanahan
Eugene Shapiro
Anurag Sharma
Paul Shekelle
Kenneth Sherr
George Siberry
Mark Siedner
Robert Siliciano
Donald Silverberg
Julie Simpson
Jerome Singh
Chris Smith
Elizabeth Smith
Douglas Smith
Helen Soberg
Ole Søgaard
Annette Sohn
Claudia Sommer
Dahye Song
Henrik Sørensen
Michael Spagat
David Spencer
Steven Spitalnik
Adam Spivak
Devi Sridhar
Christine Staatz
Emmanuel Stamatakis
Richard Steen
Deborah Stein
Michael Steinman
Richard Steketee
Olof Stephansson
Jonathan Sterne
William Stewart
Robert Stewart
Ewout Steyerberg
Sarah Stock
Bradley Stoner
Bjørn Strand
Hendrik Streeck
Nathalie Streichenberger
Susan Sturgeon
Eric Suba
Ramnath Subbaraman
Christopher Sudfeld
Cris Sullivan
Qi Sun
Sudha Sundar
Jeremy Sussman
Richard Sutton
Ronald Swanstrom
Dallas Swendeman
Daniel Swerdlow
Matthis Synofzik
Julie-An Talano
Wing Hung Tam
Joel Tarning
Hugh Taylor
Melanie Taylor
Kok Keng Tee
Parisa Tehranifar
Olli Tenovuo
Fern Terris-Prestholt
Harsha Thirumurthy
Brett Thombs
Jessica Thomson
Claire Thorne
Jim Thornton
Kamala Thriemer
Rebecca Thurston
Mark Thursz
Matthew Todd
Francine Toye
Lydie Trautmann
Iñaki Troconiz
Claudia Trudel-Fitzgerald
Alexander Tsai
Ashleigh Tuite
Janet Turan
Grace Turner
Katherine Turner
Thorkild Tylleskär
Magnus Unemo
Stefan Unger
Alexander Upfill-Brown
Omolara Uwemedimo
Salvatore Vaccarella
Masoud Vaezghasemi

Alex Valadka
Janneke van de Wijgert
Meta van den Heuvel
Saskia van der Kam
Daniëlle van der Windt
Boris van Passel
Joost van Rosmalen
Wesley Van Voorhis
Linos Vandekerckhove
Russel VanDyke
Francis Varaine
Apostolos Veizis
Ruud Veldhuizen
Christos Venetis
Atheendar Venkataramani
Donata Vercelli
Sten Vermund
Andy Vernon
Cecile Viboud
Peter Vickerman
Petter Viksveen
Diego Villar
Michael Von Korff
Lorenz von Seidlein
Trudy Voortman
Bradley Wagner
Paul Walker
Rebecca Walker
Jennifer Walker
Patrick Walker
Sharon Walmsley
Yanzhong Wang
Hui Wang
Peter Ward
James Wason
Heather Watts
J. Todd Weber
Daniel Weinberger
David Weiner
Sheri Weiser
Paul Welsh
Stephen Weng
Rudi Westendorp
David Whiley
Martin White
Peter White
Michael White
Teodora Wi
Ellen Wiewel
Marian Willinger
Charles Willis-Owen
Nick Wilson
Peter Wilson
Christopher Wisniewski
Jean Woo
Sarah Woodhall
Mark Woodward
Peter Wright
Zunyou Wu
Ming Xian
Jingwu Xie
Maria Xiridou
Seungmi Yang
Haifeng Ye
Jennifer Yokoyama
Robin Young
A. Yu
Emily Zabor
Jean-François Zagury
Arianna Zanolini
Jiaying Zhao
Pingyu Zhou
Cathy Zimmerman
Franklin Zimring
Marcel Zwahlen
